# (S)WASH-D for Worms: A pilot study investigating the differential impact of school- versus community-based integrated control programs for soil-transmitted helminths

**DOI:** 10.1371/journal.pntd.0006389

**Published:** 2018-05-03

**Authors:** Naomi E. Clarke, Archie C. A. Clements, Salvador Amaral, Alice Richardson, James S. McCarthy, John McGown, Stuart Bryan, Darren J. Gray, Susana V. Nery

**Affiliations:** 1 Research School of Population Health, Australian National University, Canberra, Australia; 2 QIMR Berghofer Medical Research Institute, Brisbane, Australia; 3 Plan International Timor-Leste, Dili, Timor-Leste; 4 Cruz Vermelha Timor-Leste, Dili, Timor-Leste; Michigan State University, UNITED STATES

## Abstract

**Background:**

Soil-transmitted helminths (STH) infect nearly 1.5 billion individuals globally, and contribute to poor physical and cognitive development in children. STH control programs typically consist of regular delivery of anthelminthic drugs, targeting school-aged children. Expanding STH control programs community-wide may improve STH control among school-aged children, and combining deworming with improvements to water, sanitation and hygiene (WASH) may further reduce transmission. The (S)WASH-D for Worms pilot study aims to compare the differential impact of integrated WASH and deworming programs when implemented at primary schools only versus when additionally implemented community-wide.

**Methodology/Principal findings:**

A two-arm, non-randomized cluster intervention study was conducted. Six communities were identified by partner WASH agencies and enrolled in the study. All communities received a school-based WASH and deworming program, while three additionally received a community-based WASH and deworming program. STH infections were measured in school-aged children at baseline and six months after deworming. Over 90% of eligible children were recruited for the study, of whom 92.3% provided stool samples at baseline and 88.9% at follow-up. The school WASH intervention improved school sanitation, while the community WASH intervention reduced open defecation from 50.4% (95% CI 41.8–59.0) to 23.5% (95% CI 16.7–32.0). There was a trend towards reduced odds of *N*. *americanus* infection among children who received the community-wide intervention (OR 0.42, 95% CI 0.07–2.36, *p* = 0.32).

**Conclusions:**

This pilot study provides proof of principle for testing the hypothesis that community-wide STH control programs have a greater impact on STH infections among children than school-based programs, and supports the rationale for conducting a full-scale cluster randomized controlled trial. High recruitment and participation rates and successful implementation of school WASH programs demonstrate study feasibility and acceptability. However, eliminating open defecation remains a challenge; ongoing work is required to develop community sanitation programs that achieve high and sustainable latrine coverage.

**Trial registration:**

Australian New Zealand Clinical Trials Registry (ANZCTR) ACTRN12615001012561

## Author summary

Soil-transmitted helminth (STH) infections are widespread globally, and their health impacts include poor growth and impaired cognitive development in children. STH control programs therefore usually focus on delivering deworming medications to school-aged children. However, expanding such programs to the whole community may result in larger benefits for children. Additionally, using water, sanitation and hygiene (WASH) interventions has been encouraged, to augment deworming programs by reducing environmental transmission. We conducted a pilot study to investigate the impact of an integrated WASH and deworming program implemented at primary schools only with a similar program implemented community-wide. Results confirm that the study procedures were feasible and acceptable to participants. Furthermore, results provide preliminary evidence that a community-wide control program will result in greater reductions in STH prevalence among school-aged children, compared to a school-based program.

## Introduction

Soil-transmitted helminthiases–caused by roundworm (*Ascaris lumbricoides*), hookworm (*Necator americanus*, *Ancylostoma duodenale*, and *Ancylostoma ceylanicum*) and whipworm (*Trichuris trichiura*)–constitute the world’s most common parasitic diseases of humans, with an estimated 1.45 billion people affected globally [1]. Soil-transmitted helminth (STH) infections are diseases of poverty, spread through fecal contamination of soil in areas that lack adequate water, sanitation and hygiene (WASH) [2].

Heavy STH infections are associated with iron-deficiency anaemia, poor growth, and impaired cognitive development [2], despite recent controversy over the benefits of treatment in terms of morbidity reversal [3]. Children are believed to suffer the majority of STH-associated morbidity, partly due to a peak in *A*. *lumbricoides* and *T*. *trichiura* prevalence and infection intensity among school-aged children [2]. Although hookworm tends to increase in prevalence towards adulthood, children and pregnant women are most at risk of the adverse effects of iron-deficiency anaemia [4].

Regular distribution of anthelminthic drugs, aiming to reduce morbidity, is the cornerstone of STH control programs, as advocated by the World Health Organization (WHO) [5]. Current guidelines focus on distribution of these medications to school-aged children, through school-based deworming programs, whereby deworming tablets are administered by teachers to all children, regardless of infection status [6]. Preschool-aged children, women of child-bearing age, pregnant women after the first trimester, and adults in high-risk occupations have recently been recommended as additional targets, although no clear guidelines have been implemented to guide distribution mechanisms to reach these groups [5, 7, 8]. The 2020 WHO target for STH control is regular deworming of 75% of at-risk children [9]; with over 550 million children treated in 2015, progress is on track to achieve this goal [10].

In the context of substantial global attention to neglected tropical disease (NTD) control, there is evolving interest in the most effective and sustainable ways to control STH infections. Integration of deworming programs with WASH improvements has been advocated, and included in recent WHO policies [11–13]. By reducing both environmental contamination with helminth eggs and larvae, and human exposure to these infective stages, WASH interventions may be essential for achieving long-term control of STH. Meta-analyses of mainly observational studies of WASH components suggest a reduction in STH infection [14, 15], and a cluster randomized controlled trial (RCT) examining a school-based WASH intervention reported reduced *A*. *lumbricoides* prevalence [16]. School-based health education programs have also been shown to reduce STH infections and intensity [17, 18]. However, RCTs of community-based WASH interventions have failed to show an impact on STH infection, potentially due to low intervention uptake and use [19, 20].

Expanding the target population of deworming programs to improve STH control has also been advocated [21]. In a recent meta-analysis, it was concluded that expanding deworming programs to whole communities would likely lead to additional benefits for children, indicating a significantly greater reduction in STH prevalence following community-wide deworming compared to child-targeted deworming [22]. With increasing interest in STH transmission interruption, mathematical modelling and cost-effectiveness studies have also highlighted the importance of expanding beyond school-based deworming [23–26]. In particular, it has been shown that the elimination of hookworm, where adults act as a substantial reservoir, will only be possible if mass drug administration campaigns include adults [27].

No prospective studies have directly compared school-based STH control programs with community-wide STH control programs, although a trial is currently underway in Kenya [28]. We conducted a pilot study, in preparation of a fully-powered cluster RCT, to compare the impact of school-based and community-wide integrated control programs, consisting of both WASH and deworming, on STH infections among school aged children.

The specific objectives of this study are as follows:

1. To assess the feasibility and acceptability of conducting a trial studying the impact of school- vs community-based distribution of deworming medications along with WASH programs, by examining study participation and recruitment rates and intervention outputs;
2. To establish “proof of principle” (preliminary evidence) to support our hypothesis that a community-wide STH control program is more effective than an exclusively school-based approach in reducing STH infections and infection intensity in school-aged children, by comparing estimates of intervention impact.

## Methods

### Study design and participants

This was a two-arm, non-randomized cluster intervention trial (see S1 Checklist) [29]. Six primary schools (clusters) were included in the pilot study. Three received only a school-based WASH and deworming program (control arm), while three additionally received a community-based WASH and deworming program in the community where the school was located (intervention arm).

The study was undertaken in Aileu and Manufahi municipalities of Timor-Leste between April 2015 and June 2016. Schools and their communities were identified in consultation with partner WASH agencies, who were responsible for implementing the WASH interventions. Different partner agencies were used for each of the two study arms, due to logistical and timing constraints that rendered it impossible to identify a partner agency with capacity to implement both study arms in the pilot phase of the study. Cruz Vermelha Timor-Leste (CVTL; Timor-Leste Red Cross Society) implemented the WASH programs in the control clusters, while Plan International implemented the WASH programs in the intervention clusters.

Participants in both arms of the study were school-aged children. All children attending the local primary school were eligible for participation in the study; there were no exclusion criteria.

### Ethics statement

The protocol for this pilot study was developed to reflect that planned for a large scale cluster RCT, and has been published previously [30]. The study is registered with the Australian New Zealand Clinical Trials Registry, registration number ACTRN12615001012561 (see S1 Protocol).

This study received ethical approval from the Human Research Ethics Committees at Australian National University (2015/111) and the Timor-Leste Ministry of Health (2015/196). The study was explained to students and their parents or guardians at a meeting at the primary school; written and schematic information sheets were provided. Written informed consent was obtained from parents or guardians via signature or ink thumbprint.

### Study interventions

The study interventions are described in detail in the protocol paper [30]. Briefly, following baseline data collection, all schools received a WASH program, consisting of: (a) provision of access to a reliable source of protected water for use by the school, achieved through constructing tapped water tanks gravity-fed from protected local springs; (b) construction of pour-flush concrete-lined pit latrines with concrete superstructures for use by students and teachers, following the Timor-Leste “WASH in Schools” guidelines [31]; and (c) hygiene education sessions conducted at school, emphasizing the importance of using latrines, handwashing with soap at key times, and keeping the environment clean, achieved through the use of flipcharts, posters and participatory demonstrations.

In the intervention arm, a WASH program was also implemented in the community where the school was located. This consisted of: (a) a sanitation intervention, aiming to increase the number of household latrines through a process known as Community-Led Total Sanitation, which challenges community members to reflect on their defecation practices and encourages them to take responsibility for building household latrines [32]; and (b) community-wide hygiene education, emphasizing key health promotion messages as above, conducted by WASH agency staff at a household level. The three intervention communities had previously received a community-level water intervention, which involved construction of tap stands supplied by gravity-fed systems from protected springs, providing access to a reliable source of protected water for use by all community members.

Following completion of the WASH intervention (defined as completed functional school latrines and 80% community latrine coverage, as reported by partner WASH agencies), the research team delivered deworming medication. In both study arms, deworming medication was delivered by study fieldworkers at school to all primary school children. In the intervention clusters, house-by-house delivery of deworming medications was additionally undertaken by fieldworkers to administer treatment to all community members, excluding children under 12 months of age and pregnant women in the first trimester. A single dose of albendazole (400mg) was used, as per WHO guidelines [5]. All tablets were taken under direct observation of fieldworkers. Study follow-up was conducted six months following albendazole distribution.

Due to the nature of the interventions, participants and intervention implementers (research team and WASH agency staff) could not be blinded to the study arm assignment.

### Data collection

At baseline and six month follow-up, students and their parents completed questionnaires, administered as interviews by trained local fieldworkers at the primary schools. Students answered questions relating to their defecation and hygiene practices. Parents answered questions relating to household water source, assets, education and occupation.

Stool samples were collected from participating students at baseline and at follow-up for diagnosis of STH infections. Children were educated on how to provide a stool sample and asked to bring a sample from the following morning to school. Aliquots of 2–3 grams were preserved immediately upon receipt of the samples with 5mL of 5% potassium dichromate, and transported at room temperature to QIMR Berghofer Medical Research Institute in Brisbane, Australia. Samples were analysed using a quantitative polymerase chain reaction (qPCR) technique, which involved DNA extraction, followed by running a real-time multiplex PCR to detect and quantify STH (*Ascaris* spp., *N*. *americanus*, *Ancylostoma* spp., *T*. *trichiura*), as described previously [33]. Laboratory staff were not aware of the study arm to which participants belonged.

At both study time points, children underwent anthropometric measurement of height (to the nearest 0.1cm) and weight (to the nearest 0.5kg); a fingerprick blood sample was also collected to measure haemoglobin using a portable analyser (Hb 201+, HemoCue, Angelholm, Sweden).

### Outcomes

Primary outcomes related to study feasibility and acceptability, and secondary outcomes related to study intervention impact (see Box 1).

**Box 1. Study outcomes (measured at baseline and six month follow-up).**

**Primary outcomes**

Proportion of eligible children for whom parental informed consent is gained

Proportion of eligible children who provide stool samples

Proportion of eligible children who complete questionnaires

Proportion of eligible children who undergo measurement of height, weight and haemoglobin

Proportion of eligible children and community members who receive albendazole

Time taken to complete school WASH interventions in all clusters

Time taken to achieve 80% household latrine coverage in intervention clusters

Proportion of schools and households with functional and clean latrines

Proportion of children who report practicing open defecation

Proportion of schools with handwashing stations

Proportion of children who practise handwashing with soap at critical times

**Secondary outcomes**

Infection with *Ascaris* spp., *N*. *americanus*, *Ancylostoma* spp., and *T*. *trichiura*

Infection intensity category of *Ascaris* spp., *N*. *americanus*, *Ancylostoma* spp., and *T*. *trichiura*

Anaemia

Malnutrition indicators: stunting, thinness, and underweight

Infection intensity categories were defined as “higher intensity”, “lower intensity”, and “no infection”, and were used to examine relative changes in infection intensity over time. Samples were categorized based on the cycle threshold (Ct) values obtained by qPCR; lower Ct values reflect higher infection intensity. We determined the median Ct value for all positive samples at baseline, and classified individuals with Ct values lower than baseline median as “higher intensity” infections, and those with Ct values higher than baseline median as “lower intensity” infections (see S1 Appendix). These categories were not intended to correspond with WHO thresholds for heavy, moderate and light-intensity infections because PCR-based values have not yet been identified that accurately correspond with these thresholds.

Anaemia was diagnosed from altitude-adjusted haemoglobin measurements using WHO thresholds [34]. The 2006 WHO database for child growth standards were used to calculate *Z*-scores for the following anthropometric indices: weight-for-age (for children aged ≤10 years only), height-for-age, and body mass index-for-age. These *Z*-scores were used to determine presence of underweight, stunting, and thinness, respectively, with scores below –2 indicative of malnutrition [35].

### Statistical analysis

For the primary outcomes, descriptive statistics were used to determine the proportion of eligible participants who gave informed consent and participated in study procedures, as well as outcomes relating to completion and coverage of the WASH interventions. WASH outputs were compared between intervention and control arms using a difference in differences (DID) approach, where DID = (Intervention_Follow-up_−Intervention_Baseline_)–(Control_Follow-up_−Control_Baseline_).

For unadjusted analysis of secondary outcomes, the proportions of children with each STH infection, higher-intensity infection, anaemia, stunting, thinness and underweight, were compared between study arms using a difference in differences approach. We also calculated the prevalence reduction ([Prevalence_Baseline_−Prevalence_Follow-up_] / Prevalence_Baseline_) of each STH infection, with confidence intervals calculated using a bootstrap resampling method with 2000 replicates.

Generalized linear mixed models [36] were then used to calculate adjusted odds ratios (OR) for infection presence and intensity group, comparing study arms at follow-up. Bernoulli logistic regression was used for infection presence, and ordinal logistic regression for infection intensity group (no, lower-intensity, and higher-intensity infection). Age, sex and baseline infection status were entered as fixed effects. Due to discordances between study arms in terms of hygiene behaviour and access to improved water at baseline, handwashing after defecation and access to improved water were also included as fixed effects. School was entered as a random effect to account for clustering. Due to very low baseline prevalence of *Ancylostoma* spp. and *T*. *trichiura*, and a highly imbalanced baseline prevalence of *Ascaris* spp. across study arms, generalized linear mixed models were run only for *N*. *americanus* infections. All analyses were conducted using Stata version 14.1 (College Station, TX, USA).

## Results

### Baseline characteristics

**Fig 1:**
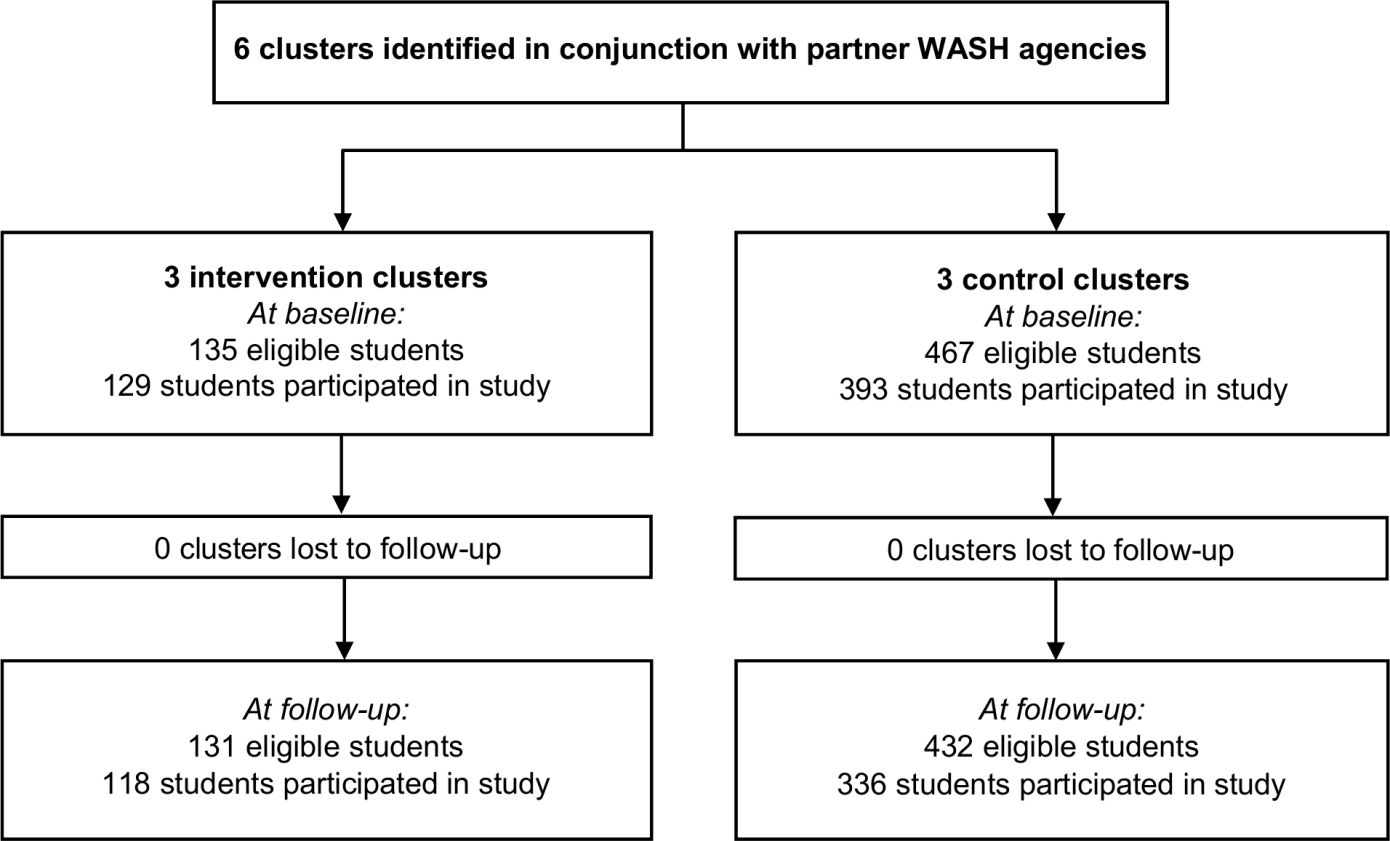
CONSORT flow diagram of the pilot study. Study participation is defined as providing a questionnaire and/or stool sample.

At study baseline, informed consent was obtained for 522 students across the six participating schools. Owing to size differences in the schools, there were three times as many participants in the control arm compared to the intervention arm. The CONSORT trial profile [37] is shown in Fig 1.

Baseline characteristics of participating students and schools, including baseline STH infections and morbidity indicators, are shown in Table 1. Age and sex were balanced across study arms. The prevalence of *Ascaris* spp. was significantly imbalanced between the two study arms, with baseline prevalence of 48.7% (95% confidence interval (CI) 43.6–53.8) in the control arm vs 7.6% (3.8–14.4) in the intervention arm (*p =* 0.007). The prevalence of *N*. *americanus* was more balanced across study arms (13.7% vs 15.1%; *p* = 0.956), while the prevalence of *Ancylostoma* spp. and *T*. *trichiura* was very low across both study arms. Stunting and underweight were each present in more than half of the study participants.

**Table 1:** Baseline characteristics of study participants.

	Control arm	Intervention arm
**Demographics**	*n = 393*	*n = 129*
Female sex, n (%)	195 (52.7%)	58 (45.0%)
Mean (range) age in years	9.64 (4.2–17.1)	9.05 (5.2–15.8)
**STH infections**	*n = 372*	*n = 110*
*Ascaris* spp. infections	48.7% (43.6–53.8)**	7.6% (3.8–14.4)**
*Ascaris* spp. higher-intensity infections	27.3% (21.1–30.0)	0.9% (0.1–6.5)
*N*. *americanus* infections	13.7% (10.6–17.6)	15.1% (9.4–23.3)
*N*. *americanus* higher-intensity infections	7.3% (5.0–10.4)	6.6% (3.2–13.3)
*Ancylostoma* spp. infections	1.1% (0.4–2.8)	0
*T*. *trichiura* infections	2.2% (1.1–4.3)	1.9% (0.5–7.3)
**Hematological parameters**	*n = 381*	*n = 116*
Anaemia	12.6% (9.6–16.3)	4.3% (1.8–10.0)
**Growth parameters (all age groups)**	*n = 382*	*n = 124*
Stunting	51.7% (46.6–56.8)	62.1% (53.2–70.2)
Thinness	25.5% (21.3–30.1)	42.7% (34.3–51.6)
**Growth parameters (age ≤10 years only)**	*n = 225*	*n = 86*
Underweight	53.3% (46.7–59.8)	65.1% (54.4–74.5)
**School-level variables**	*n = 3*	*n = 3*
Mean number of students (SD)	175.3 (97.3)*	52.0 (16.4)*
Mean proportion girls (SD)	50.1 (3.0)	48.5 (10.3)
Mean pupil/teacher ratio (SD)	26.4 (2.3)	18.6 (8.5)

Unless otherwise indicated, results are shown as: proportion (95% confidence interval)

Significant difference between study arms:

* p<0.05

** p<0.01

### Study recruitment and participation

Over 90% of students who were present during the research team visits were recruited (i.e., parental informed consent obtained) at each study time point; there were no refusals of consent. At baseline, among the 522 students for whom informed consent was gained, 92.3% provided stool samples, 100% completed questionnaires, 95.4% provided blood samples, and 96.9% had their height and weight measured. Participation rates remained similarly high at follow-up (see Table 2). There were no differences in participation rates between study arms at either time point. Following the implementation of the WASH programs, albendazole was distributed to 89.4% of eligible schoolchildren, and in the intervention arm of the study, to 395 out of 471 (83.9%) eligible community members.

**Table 2:** Recruitment and participation in the pilot study.

	Baseline		Follow-up	
	Control	Intervention	Control	Intervention
Eligible students (n)^a^	467	135	432	131
Students present, n (%)	432 (92.5%)	131 (97.8%)	378 (87.5%)	124 (94.7%)
Consent obtained, n (%)^b^	393 (90.5%)	129 (98.5%)	341 (90.2%)	120 (96.8%)
Provided stool, n (%)^c^	372 (94.7%)	110 (85.3%)	303 (88.9%)	107 (89.2%)
Completed questionnaire, n (%)^c^	393 (100%)	129 (100%)	336 (98.5%)	118 (98.3%)
Provided blood sample, n (%)^c^	382 (97.2%)	116 (89.9%)	324 (95.0%)	112 (93.3%)
Height/weight measured, n (%)^c^	382 (97.2%)	124 (96.1%)	326 (95.6%)	116 (96.7%)
Albendazole taken, n (%)^d^	393/444 (88.5%)	124/134 (92.5%)	359/432 (83.1%)	120/131 (91.6%)

^a^ Eligible students defined as all those enrolled to attend the primary school

^b^ Proportion calculated out of total students present

^c^ Proportion calculated out of total students for whom informed consent was obtained

^d^ Proportion calculated out of total students enrolled in primary school at time of albendazole distribution

### WASH indicators

Community latrine coverage reached 80% within three months in all intervention communities. The school WASH program was completed within five months in five schools. In the remaining school, it took seven months to complete, and in order to treat participating children before school holidays commenced, albendazole was administered before school latrine construction was completed. The latrines were completed by the time children returned to school six weeks later.

Outcomes relating to the WASH intervention are depicted in Table 3. The school WASH intervention resulted in all schools having access to handwashing stations and functional latrines, with a mean pupil-to-latrine ratio of 25.4 students in the control arm and 26 students in the intervention arm. All schools had separate toilets for male and female students.

**Table 3:** WASH infrastructure and behaviour at baseline and follow-up.

Variable	Baseline		Follow-up	
	Control	Intervention	Control	Intervention
**School-level variables**	*n = 3*	*n = 3*	*n = 3*	*n = 3*
Schools with functional latrines, n (%)	0	0	3 (100%)	3 (100%)
Mean (SD) number of pupils per latrine	-	-	25.4 (10.6)	26.0 (8.2)
Schools with handwashing stations, n (%)	0	0	3 (100%)	3 (100%)
**Individual-level variables**^**a**^	*n = 393*	*n = 129*	*n = 329*	*n = 119*
Students reporting presence of household latrine	67.4% (62.6–71.9)	65.9% (60.7–76.7)	76.4% (71.5–80.8)	84.9% (77.2–90.3)
Students reporting open defecation	58.5% (53.6–66.3)	50.4% (41.8–59.0)	45.9% (40.6–51.3)	23.5% (16.7–32.0)
Students with access to improved water^b^	53.1% (48.5–58.4)	83.7% (76.2–89.2)	69.2% (63.7–74.1)	86.1% (78.4–91.4)
Students reporting use of soap when washing hands	87.3% (83.6–90.2)	72.9% 64.5–79.9)	91.2% (87.6–93.8)	88.2% (81.1–92.9)
Students reporting handwashing after defecation	59.0% (54.1–63.8)	38.0% (30.0–46.7)	70.8% (65.7–75.5)	61.3% (52.3–69.7)
Students reporting handwashing before eating	30.8% (26.4–35.5)	14.7% (9.6–22.0)	44.1% (38.8–49.5)	41.2% (32.6–50.3)

^a^ Individual level variables are shown as proportion (95% confidence interval).

^b^ Access to improved water defined as main household water source being either piped water, protected spring, or protected dugwell.

Baseline household latrine coverage was higher than expected in both study arms; approximately two thirds of children reported presence of a household latrine. However, open defecation was reported by 58.5% (53.6–66.3) children in the control arm and 50.4% (41.8–59.0) in the intervention arm at baseline. At follow-up, the proportion of children reporting household latrines increased to 76.4% (71.5–80.8) in the control arm and 84.9% (77.2–90.3) in the intervention arm, with the difference in differences (DID) between intervention and control arms of 6.6% (*p* = 0.211). Children practicing open defecation decreased to 45.9% (40.6–51.3) in the control arm, and 23.5% (16.7–32.0) in the intervention arm, with DID of -14.2% (*p* = 0.032).

At study baseline, more children in the intervention arm had access to an improved water source, compared to the control arm (83.7% vs 53.1%); this difference persisted at study follow-up (86.1% vs 69.2%). On the other hand, children in the control arm displayed better hygiene behaviour than those in the intervention arm at baseline, with a higher proportion of students reporting use of soap (87.3% vs 72.9%), handwashing after defecation (59.0% vs 38.0%), and handwashing before eating (30.8% vs 14.7%). Following the WASH program, hygiene behaviour improved in both study arms. DID between intervention and control arms was 11.5% for use of soap (*p* = 0.150), 11.6% for handwashing after defecation (*p* = 0.373) and 13.2% for handwashing before eating (*p* = 0.219).

### Intervention impact

**Fig 2:**
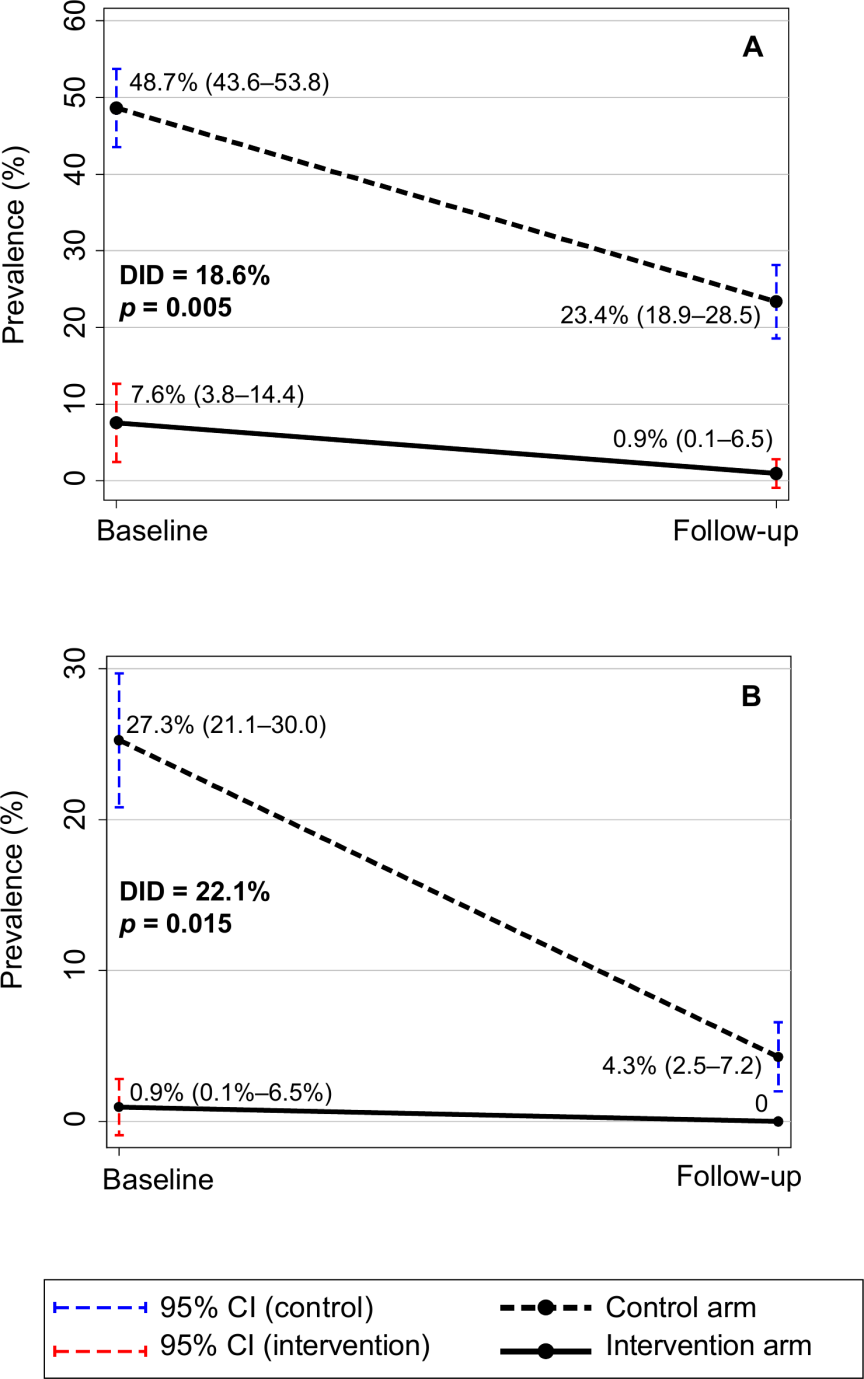
**Prevalence of (A) infection and (B) higher-intensity infection with *Ascaris* spp., before and six months following the study intervention.** *P* values are based on logistic regression models comparing intervention and control arms, accounting for school-level clustering. CI = confidence interval; DID = difference in differences between intervention and control arms.

**Fig 3:**
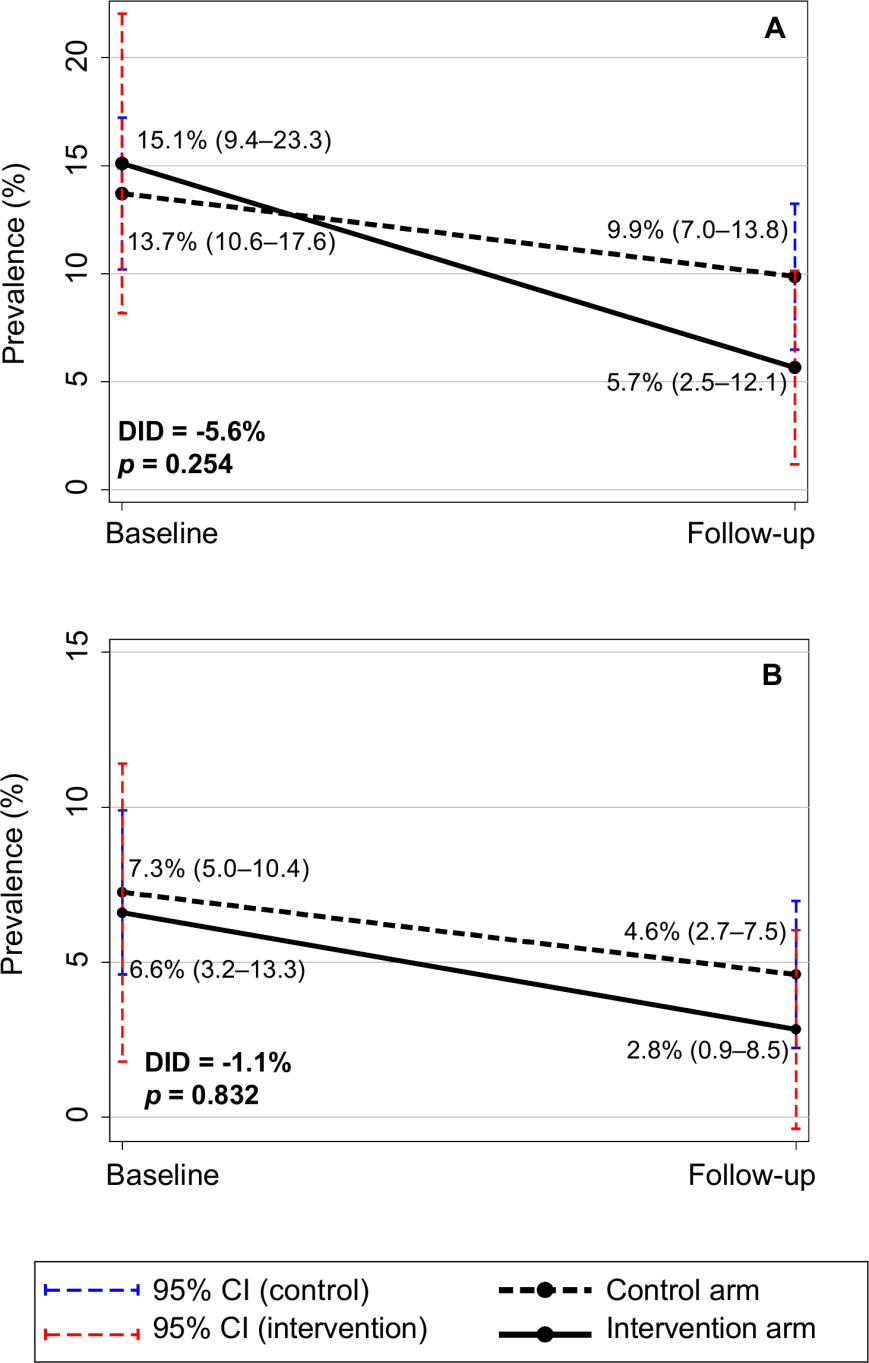
**Prevalence of (A) infection and (B) higher-intensity infection with *N*. *americanus*, before and six months following the study intervention.** *P* values are based on logistic regression models comparing intervention and control arms, accounting for school-level clustering. CI = confidence interval; DID = difference in differences between intervention and control arms.

As shown in Fig 2 and S1 Table, six months following the WASH and deworming intervention, the prevalence of *Ascaris* spp. decreased from 48.7% (43.6–53.8) to 23.4% (18.9–28.5) in the control arm and from 7.6% (3.8–14.4) to 0.9% (0.1–6.5) in the intervention arm, representing a prevalence reduction of 52.0% (95% CI 45.2–70.0) in the control arm and 88.2% (95% CI 70.2–100.0) in the intervention arm. The crude DID between intervention and control arms was 18.6% (*p =* 0.005), reflecting the significantly higher baseline prevalence in the control arm. *N*. *americanus* prevalence decreased from 13.7% (10.6–17.6) to 9.9% (7.0–13.8) in the control arm, and from 15.1% (9.4–23.3) to 5.7% (2.5–12.1) in the intervention arm, representing a prevalence reduction of 27.7% (95% CI 12.7–40.7) in the control arm and 62.3% (95% CI 54.9–67.4) in the intervention arm (see Fig 3 and S1 Table). The crude DID between intervention and control arms was -5.6% (*p* = 0.254). The prevalence of higher-intensity infections also decreased for both STH.

Morbidity indicators remained similar across the six months of the study (S2 Table). There were no significant DID between study arms in terms of nutritional indicators or hematological parameters.

The results of the generalized linear mixed models used to assess impact of the intervention on *N*. *americanus* infection are shown in Table 4. The odds of *N*. *americanus* infection at follow-up were 58% lower in the intervention arm compared to the control arm (OR 0.42, 95% CI 0.07–2.36), and the odds of higher-intensity infection were 57% lower (OR 0.43, 95% CI 0.08–2.37), although these did not reach statistical significance. Males had increased odds of infection (OR 2.92, 95% CI 1.07–7.95). Children who had *N*. *americanus* infection at baseline had significantly increased odds of infection at follow-up (OR 10.20, 95% CI 3.68–28.27). Similarly, those with a higher-intensity infection at baseline had significantly increased odds of higher-intensity infection at follow-up (OR 15.57, 95% CI 5.15–47.06).

**Table 4:** Results of generalized linear mixed models showing effect estimate of the study intervention and other covariates on *N*. *americanus* prevalence and intensity.

Variable	*N*. *americanus* infection			*N*. *americanus* higher-intensity infection		
	OR	95% CI	*p* value	OR	95% CI	*p* value
Study intervention^a^	0.42	0.07–2.36	0.32	0.43	0.08–2.37	0.33
Age in years	1.05	0.85–1.31	0.63	1.13	0.91–1.40	0.26
Male sex^b^	2.92	1.07–7.95	0.04	2.45	0.91–6.58	0.08
Handwashing after defecation^c^	0.55	0.21–1.45	0.23	0.58	0.22–1.55	0.28
Access to improved water^d^	0.51	0.20–1.32	0.17	0.59	0.23–1.50	0.27
*N*. *americanus* infection at baseline^e^	10.20	3.68–28.27	<0.01	-	-	-
*N*. *americanus* intensity group at baseline^e^						
Lower intensity	-	-	-	5.17	1.35–19.84	0.02
Higher intensity	-	-	-	15.57	5.15–47.06	<0.01
**Random effects variance (95% CI)**						
School	0.48 (0.06–4.03)			0.48 (0.06–3.89)		

CI = confidence interval; OR = odds ratio. Reference groups are as follows

^a^ Control group

^b^ Female sex

^c^ Does not wash hands after defecation.

^d^ No access to improved water. Access to improved water is defined as main household water source being either piped water, protected spring, or protected dugwell.

^e^ No infection at baseline.

## Discussion

To our knowledge, this pilot study represents the first direct comparison of the impact of integrated school-based versus community-wide control programs on STH infection in school-aged children. The integrated STH control programs in our pilot study consisted of distribution of anthelminthic medications, regardless of infection status, complemented by WASH improvements, in line with recent WHO recommendations [38].

We achieved high rates of both parental informed consent and participation of school-aged children in all aspects of the study. Participation rates were above 80% for all study procedures (completing questionnaires, providing stool samples, undergoing anthropometric measurement, providing a fingerprick blood sample, and taking albendazole), confirming the acceptability of the study methods and procedures. There were no refusals of parental informed consent, likely because deworming medications are understood to benefit children, and WASH improvements to benefit both children and the wider community. Child refusal to participate in any component of the study was low. In particular, stool samples were collected from a high proportion of participating children (92.3% at baseline and 88.9% at follow-up), suggesting that our results are likely representative of the population STH prevalence in school-aged children.

The school WASH programs were successful in improving school sanitation, handwashing facilities, and hygiene behaviour among school-aged children. The community-wide WASH program reduced the practice of open defecation among school-aged children, such that prevalence of open defecation was significantly lower in the intervention arm compared to the control arm at follow-up. Importantly, the aim of community WASH programs is to eliminate open defecation, and in this respect, the community-wide program implemented in our study did not achieve “open defecation free” status. Nearly 25% of children in the intervention arm still reported that they practised open defecation at study follow-up. These results are consistent with other community-based trials where WASH programs have been implemented and where health improvements have not been detected as a result [19, 20].

The integrated WASH and deworming interventions reduced STH prevalence and intensity in both study arms. Morbidity outcomes, including anaemia, stunting, thinness and underweight, were mainly measured to establish feasibility; significant changes were not anticipated within a six-month time frame. As expected, these indicators remained similar across the six-month study period.

Importantly, results of the pilot study showed a 58% reduction in odds of *N*. *americanus* infection and 57% reduction in odds of higher-intensity infection in the intervention arm (community-wide control program), compared to the control arm (school-based control program). In the context of a small pilot study, these results did not reach statistical significance. This was to be expected, because the pilot study was not powered to detect such differences between study arms. However, these results provide preliminary evidence and proof of principle for testing our hypothesis that a community-wide control program will be more effective at reducing STH infections in children than a school-based control program. Our findings agree with a recent meta-analysis, as well as with mathematical modelling studies, highlighting the additional benefits of expanding STH control programs community-wide [22–26].

### Study limitations and lessons learned

This pilot study highlighted several important issues for consideration during planning and implementation of a larger-scale trial. Firstly, a significant limitation of this pilot study was the non-randomization of study schools. Randomization was not possible because we were unable to identify a partner agency who was able to implement the WASH interventions in both arms of the study within required timeframes. Therefore, two different partner agencies were used (one for each study arm), who worked in two different municipalities of Timor-Leste. Non-randomization led to a number of discrepancies between study arms at baseline, including school size, *Ascaris* spp. prevalence, and hygiene behaviour. Hygiene behaviour was significantly better among children in the control arm at baseline. Given previous findings that school-based health education programs may significantly reduce STH reinfection [17], this could bias results against showing an intervention impact. Therefore, we adjusted for baseline hygiene behaviour in our final analysis. The highly discordant baseline prevalence of *Ascaris* spp. made interpretation of intervention impact on infection with this parasite impossible, limiting our analysis to *N*. *americanus*. Such discrepancies would likely be avoided in a fully-powered study that was adequately randomized.

We collected information on household WASH conditions using self-reported data from children and parents in interviews conducted at the school. This approach may result in a response bias; participants may be more likely to report favourable answers if they are reluctant to give negative feedback (courtesy bias), or on the other hand, they may report less favourable answers if they anticipate NGO intervention. In a larger trial, household inspections should be carried out in order to improve accuracy of the measurement of WASH conditions, both before and after intervention implementation.

The school WASH programs were commenced mid-way through the school year, and due to school holiday timing, albendazole was distributed before latrines were completed in one school in the intervention arm. This could have led to higher reinfection rates and biased results against showing an intervention effect. A fully-powered trial should be implemented such that the WASH intervention is conducted close to the beginning of the school year, and it is crucial to ensure that partner WASH agencies have capacity to complete their interventions in a timely and simultaneous manner. Successful study implementation will require co-operation and regular liaison between the research team, WASH agencies, school and community leaders, and the Ministry of Education.

We observed a higher than expected baseline coverage of household latrines; as a result, improvements to sanitation coverage achieved through the community WASH program were modest. Additionally, water improvements were implemented in the intervention communities prior to study baseline; this was adjusted for in our analysis. Both of these factors may have decreased the likelihood of the WASH intervention reducing STH transmission. In a larger trial, a thorough assessment of potential study communities should be conducted to ensure that the communities are appropriate for a WASH intervention, with clearly defined eligibility criteria, such as a cut-off for baseline latrine coverage and water availability.

Finally, although we observed a greater than 50% decrease in open defecation following the community WASH program, the goal of eliminating the practice of open defecation was not achieved. Our pilot study highlighted the difficulty in achieving high and sustainable latrine coverage that has been seen previously [19, 20], and the need for ongoing studies within the WASH sector to identify and assess innovative strategies for improving and sustaining the coverage and use of sanitation facilities. When designing a larger-scale trial, rigorous discussion and planning with partner agencies should be undertaken in an effort to maximize intervention uptake and fidelity.

### Conclusions and future directions

Our results demonstrate proof of principle for testing the hypothesis that an expanded community-wide STH control program will lead to reduced STH reinfection among school-aged children compared to a school-based control program. These results highlight the feasibility and rationale for conducting a full-scale cluster RCT comparing community-wide and school-based STH control approaches to test this hypothesis, and to inform global STH control guidelines.

## Supporting information

S1 Checklist TREND checklist for reporting of non-randomized clinical trials. (PDF)

S1 Protocol Full study protocol from the Australian New Zealand clinical trial registry. (PDF)

S1 Appendix STH infection intensity categories. (PDF)

S1 Table STH infections over time. (PDF)

S2 Table Morbidity indicators over time. (PDF)
