# Supplementary material for: (S)WASH-D for Worms: A pilot study investigating the differential impact of school- versus community-based integrated control programs for soil-transmitted helminths
Source: PLoS Negl Trop Dis. 2018 May 3;12(5):e0006389. doi: 10.1371/journal.pntd.0006389 (PMC5933686; doi:10.1371/journal.pntd.0006389)
Supplement: S1 Protocol — (PDF) [file pntd.0006389.s002.pdf]

Register a trial

Acknowledgment	Step 1: Titles & IDs	Step 2: Health condition	Step 3: Intervention/exposure
Step 4: Outcomes	Step 5: Eligibility	Step 6: Study design	Step 7: Recruitment
Step 8: Funding & Sponsors	Step 9: Ethics & Summary	Step 10: Contacts	Review & Submit

**Request number**	369077
**Current page**	Review

Trial registered on ANZCTR

**Trial ID**	ACTRN12615001012561
**Ethics application status**	Approved
**Date submitted**	4/09/2015
**Date registered**	28/09/2015
**Type of registration**	Retrospectively registered

Titles & IDs

**Public title**	Should integrated deworming and water, sanitation and hygiene (WASH) programs for soil-transmitted helminth (STH) control be delivered in schools or the community? A pilot study
**Scientific title**	A pilot study comparing the impact of school- and community-based integrated water, sanitation and hygiene (WASH) and deworming programmes on soil-transmitted helminth infections in school-aged children in Timor-Leste
**Secondary ID [1]**	OPP1119041 (Grant number from Bill and Melinda Gates Foundation)
**Universal Trial Number (UTN)**	U1111-1172-9719
**Trial acronym**	(S)WASH-D for Worms pilot
**Linked study record**	

Health condition

Health condition(s) or problem(s) studied:

Soil-transmitted helminth infection - Trichuris trichiura, Ascaris lumbricoides, hookworms (Necator americanus and Ancylostoma duodenale)

Stunting

Wasting

Anaemia

Intestinal protozoa (Giardia duodenalis, Entamoeba histolytica, Strongyloides spp., Cryptosporidium spp.)

Condition category	Condition code
Infection	Other infectious diseases
Public Health	Epidemiology
Oral and Gastrointestinal	Other diseases of the mouth, teeth, oesophagus, digestive system including liver and colon

Intervention/exposure

**Study type**	Interventional
**Description of intervention(s) / exposure**	The intervention to be evaluated in this proposal will involve provision of access to improved water and sanitation and improving related hygiene practices, implemented at both a community level and a primary school level. This intervention will be implemented by non-governmental organisation Plan International in Timor-Leste. The sanitation component will involve construction of school latrines by contractors working with Plan International, as well a Community Led Total Sanitation approach. Access to an improved water supply will also be provided, and local partner NGOs will provide house-by-house education on hygiene practices, in particular hand-washing with soap at critical times. Hygiene education including posters relating to handwashing with soap will be provided to schools, and handwashing stations with soap will be constructed as part of the school latrines.<br><br>Furthermore, communities in the intervention arm of the pilot study will receive mass chemotherapy (distributed to all members of the community) with one oral tablet of albendazole 400mg, which will be

	administered once 80% of the households have sanitation (as defined by the presence of a household latrine) and the school latrines have been completed. Albendazole intake will be directly observed by the field workers delivering the tablets, who will be working under the supervision of a registered nurse.<br><br>The intervention period will continue until the school latrine construction is finished, household latrine construction is complete, hygiene promotion has been conducted in all households and mass chemotherapy has been delivered. This is estimated to take between 2-4 months.
**Intervention code [1]**	Prevention
**Intervention code [2]**	Treatment: Drugs
**Intervention code [3]**	Behaviour
**Comparator / control treatment**	Communities in the control group will be provided with access to improved water and sanitation and hygiene promotion implemented only at primary school level. This will be implemented by non-governmental organisation Cruz Vermelha Timor-Leste (CVTL), and will involve construction of school latrines, access to an improved water supply and promotion of hand washing with soap and related hygiene behaviours. This intervention will be similar to that in the intervention arm (although conducted by a different NGO) but will only be delivered to primary school children.<br><br>Furthermore, communities in the control arm of the pilot study will receive chemotherapy (distributed to school-aged children only) with one oral tablet of albendazole 400mg, which will be administered once the school latrines have been completed. Albendazole intake will be directly observed by the field workers delivering the tablets, who will be working under the supervision of a registered nurse.
**Control group**	Active

Outcomes

**Primary outcome [1]**	Cumulative incidence of of infection with A. lumbricoides, T. trichiura, N. americanus and Ancylostoma spp. (undifferentiated) in school aged children - to be assessed by both microscopy and PCR examination of stool
**Timepoint [1]**	At baseline and at follow-up six months after the distribution of albendazole
**Secondary outcome [1]**	Proportion of eligible children for whom informed consent is gained - using school records to determine number of eligible children
**Timepoint [1]**	At baseline and at follow-up six months after the distribution of albendazole
**Secondary outcome [2]**	Proportion of eligible children for whom stool samples are provided - using school records to determine number of eligible children
**Timepoint [2]**	At baseline and at follow-up six months after the distribution of albendazole
**Secondary outcome [3]**	Proportion of eligible children who complete questionnaires - using school records to determine number of eligible children
**Timepoint [3]**	At baseline and at follow-up six months after the distribution of albendazole
**Secondary outcome [4]**	Proportion of eligible children who undergo measurement of height, weight and haemoglobin - using school records to determine number of eligible children
**Timepoint [4]**	At baseline and at follow-up six months after the distribution of albendazole
**Secondary outcome [5]**	Prevalence of S. stercoralis, G. duodenalis, E. histolytica, and Cryptosporidium spp. (composite outcome) - assessed using laboratory analysis (PCR) of stool samples
**Timepoint [5]**	At baseline and at follow-up six months after the distribution of albendazole
**Secondary outcome [6]**	Mean haemoglobin concentration - measured using serum assay on a Hb201 (Hemocue) analyser device
**Timepoint [6]**	At baseline and at follow-up six months after the distribution of albendazole
**Secondary outcome [7]**	Anthropometric index weight-for-height Z-score (to identify wasting)
**Timepoint [7]**	At baseline and at follow-up six months after the distribution of albendazole
**Secondary outcome [8]**	Anthropometric index weight-for-age Z-score (to identify underweight)
**Timepoint [8]**	At baseline and at follow-up six months after the distribution of albendazole
**Secondary outcome [9]**	Anthropometric index height-for-age Z-score (to identify stunting)
**Timepoint [9]**	At baseline and at follow-up six months after the distribution of albendazole
**Secondary outcome [10]**	Mean intensity of infection (average number of eggs per gram of faeces)
**Timepoint [10]**	Six months following distribution of albendazole

Eligibility

**Key inclusion criteria**	Inclusion criteria for enrollment in the study:<br>- Child enrolled in and attending the primary school<br>- Informed consent obtained from parent/caregiver<br><br>Selection of communities for inclusion in the study:<br>- Communities were selected for inclusion in this pilot study in consultation with each partner NGO (Plan International and Cruz Vermelha Timor-Leste (CVTL))<br>- For the intervention clusters, Plan International identified three villages in which they were planning both a school- and community-based WASH programme.

	- For the control clusters, the research team and CVTL identified three schools suitable for a school-based WASH programme, located in a nearby district to the intervention communities.
**Minimum age**	1 Years
**Maximum age**	No limit
**Gender**	Both males and females
**Can healthy volunteers participate?**	Yes
**Key exclusion criteria**	Exclusion criteria for enrollment in the study:<br>- Not attending the primary school<br>- Informed consent not obtained<br><br>Exclusion criteria for receiving albendazole (including students enrolled in the study AND other members of communities in the intervention clusters):<br>- Women in the first trimester of pregnancy<br>- Children under the age of 1 year

Study design

**Purpose of the study**	Prevention
**Allocation to intervention**	Non-randomised trial
**Procedure for enrolling a subject and allocating the treatment (allocation concealment procedures)**	All children who are enrolled in and attending the primary school in each of the six communities participating in this pilot study will be eligible for inclusion in the study. Consent will be sought from parents/caregivers at a meeting which will be held at the school. Allocation is not concealed.
**Methods used to generate the sequence in which subjects will be randomised (sequence generation)**	This pilot project is not randomised. This is because the WASH intervention for each arm of the study is being performed by a different NGO, and communities participating in the study are those in which those NGOs are working.
**Masking / blinding**	Open (masking not used)
**Who is / are masked / blinded?**	
**Intervention assignment**	Parallel
**Other design features**	
**Phase**	Not Applicable
**Type of endpoint(s)**	Efficacy
**Statistical methods / analysis**	Descriptive statistics will be used to determine the proportion of eligible participants who gave informed consent, provided stool samples, completed questionnaires and underwent measurement of height and weight.<br><br>Primary and secondary outcomes will be calculated and compared across both arms of the trial using mixed effects multivariate regression models that account for clustering of participants in villages.

Recruitment

**Recruitment status**	Completed

**Date of first participant enrolment**			
**Anticipated**		**Actual**	21/05/2015

**Date of last participant enrolment**			
**Anticipated**	7/06/2016	**Actual**	2/07/2016

**Date of last data collection**			
**Anticipated**		**Actual**	3/07/2016

**Sample size**					
**Target**	475	**Current**		**Final**	557

**Recruitment outside Australia**	
**Country [1]**	Timor-Leste
State/province [1]	Aileu and Manufahi Districts

Funding & Sponsors

**Funding source category [1]**	Charities/Societies/Foundations
**Name [1]**	Bill and Melinda Gates Foundation - Grand Challenges Explorations

**Address [1]**	500 Fifth Avenue North<br>Seattle, WA 98109<br>United States of America
**Country [1]**	United States of America
**Primary sponsor type**	Individual
**Name**	Susana Vaz Nery
**Address**	ANU College of Medicine, Biology and Environment<br>The Australian National University<br>Building 62 Mills Road<br>Canberra ACT 0200
**Country**	Australia
**Secondary sponsor category [1]**	Individual
**Name [1]**	Darren Gray
**Address [1]**	ANU College of Medicine, Biology and Environment<br>The Australian National University<br>Building 62 Mills Road<br>Canberra ACT 0200
**Country [1]**	Australia
**Secondary sponsor category [2]**	Individual
**Name [2]**	Archie Clements
**Address [2]**	ANU College of Medicine, Biology and Environment<br>The Australian National University<br>Building 62 Mills Road<br>Canberra ACT 0200
**Country [2]**	Australia
**Other collaborator category [1]**	Individual
**Name [1]**	Rebecca J Traub, BSc BVMS (Hons) PhD
**Address [1]**	Faculty of Veterinary Science<br>University of Melbourne<br>Parkville VIC 3052
**Country [1]**	Australia
**Other collaborator category [2]**	Individual
**Name [2]**	James McCarthy
**Address [2]**	QIMR Berghofer Medical Research Institute<br>University of Queensland<br>Dept. of Infectious Diseases,<br>Royal Brisbane and Womens Hospital<br>Herston Rd Herston<br>QLD 4029
**Country [2]**	Australia

Ethics approval

**Ethics application status**	Approved
**Ethics committee name [1]**	The Australian National University Human Research Ethics Committee
**Ethics committee address [1]**	The Australian National University<br>Acton ACT 2601
**Ethics committee country [1]**	Australia
**Date submitted for ethics approval [1]**	20/03/2015
**Approval date [1]**	08/05/2015
**Ethics approval number [1]**	2015/111
**Ethics committee name [2]**	Cabinet for Ethics and Quality Control - Ministry of Health Timor-Leste
**Ethics committee address [2]**	Instituto National Saude<br>Comoro<br>Dili
**Ethics committee country [2]**	Timor-Leste
**Date submitted for ethics approval [2]**	13/02/2015
**Approval date [2]**	13/04/2015
**Ethics approval number [2]**	MS-INS/GDE-Peskija/II/2015/196

Summary

**Brief summary**	The current WHO strategy for control of soil-transmitted helminths (STH) is school-based targeted drug treatment focusing on school-age children. Deworming programmes with anthelminthic drugs are highly effective in reducing morbidity but rapid reinfection occurs if there is no reduction in environmental contamination with parasite infective stages. Therefore, provision of water, sanitation and hygiene (WASH) programs is of critical importance in the sustainable control of STHs. In fact, WASH programs have been shown to reduce worm infection, both when implemented at schools and in entire communities. On the other hand, recent modeling has raised questions about WHO guidelines, demonstrating limited impact from school-based delivery of interventions on community health and, importantly, STH transmission. This is contrary to the currently accepted idea that adults benefit from school-based deworming as a result of its impact on the overall intensity of transmission within the population. Therefore, when thinking of the long-term control of STH, it will be necessary to optimise strategies for deworming and WASH programs, with respect to school versus community-based delivery of interventions.<br><br>This pilot study aims to establish the feasibility of conducting a large cluster-randomised trial investigating the differential impact of school- versus community-based integrated WASH and deworming programmes. The pilot study also aims to establish "proof of principle" that a community-based intervention will be more effective than a school-based intervention at reducing STH infections in school-aged children.
**Trial website**	
**Trial related presentations / publications**	
**Public notes**	
**Private notes**	

Contacts

**Principal investigator**	
**Name**	Dr Susana Vaz Nery
**Address**	Research School of Population Health<br>The Australian National University<br>Building 62 Mills Road<br>Canberra ACT 0200
**Country**	Australia
**Phone**	+61 2 6125 0155
Fax	
**Email**	susana.nery@anu.edu.au

**Contact person for public queries**	
**Name**	Dr Susana Vaz Nery
**Address**	Research School of Population Health<br>The Australian National University<br>Building 62 Mills Road<br>Canberra ACT 0200
**Country**	Australia
**Phone**	+61 2 6125 0155
Fax	
**Email**	susana.nery@anu.edu.au

**Contact person for scientific queries**	
**Name**	Dr Susana Vaz Nery
**Address**	Research School of Population Health<br>The Australian National University<br>Building 62 Mills Road<br>Canberra ACT 0200
**Country**	Australia
**Phone**	+61 2 6125 0155
Fax	
**Email**	susana.nery@anu.edu.au

**Contact person responsible for updating information**	
**Title**	Dr
**Name**	Naomi Clarke
**Address**	Research School of Population Health<br>The Australian National University<br>Building 62 Mills Road<br>Canberra ACT 0200

**Country**	Australia
**Phone**	+61 2 6125 0155
Fax	
**Email**	naomi.clarke@anu.edu.au

Cancer fields

**Cancer stage(s)**	
**Treatment type(s)**	
**Known and possible side effect(s) for each arm of the trial (if applicable)**	
**Cost to participants**	
**Time commitment**	
**Travel**	

Copyright © Australian New Zealand Clinical Trials Registry. All rights reserved.
