# Supplementary material for: (S)WASH-D for Worms: A pilot study investigating the differential impact of school- versus community-based integrated control programs for soil-transmitted helminths
Source: PLoS Negl Trop Dis. 2018 May 3;12(5):e0006389. doi: 10.1371/journal.pntd.0006389 (PMC5933686; doi:10.1371/journal.pntd.0006389)
Supplement: S1 Appendix — (PDF) [file pntd.0006389.s003.pdf]

S1 Appendix. STH infection intensity categories

The cycle threshold (Ct) value represents the number of PCR cycles required for the fluorescence signal of the amplified DNA products to cross a set threshold value that exceeds background level. Higher quantities of DNA, reflecting more intense STH infections, will therefore result in lower Ct values. Details of the qPCR technique used in this study have been previously published [1].

The cut-offs used to determine the three infection intensity categories (no infection, lower intensity infection, and higher-intensity infection) are shown in the Table below.

Table. Ct value cut-offs for determining infection intensity categories

	**No infection**	**Lower-intensity infection***	**Higher-intensity infection***
***Ascaris* spp.**	Greater than 35	Greater than 15.885 and less than or equal to 35	Less than or equal to 15.885
Necator americanus	Greater than 35	Greater than 24.180 and less than or equal to 35	Less than or equal to 24.180

* Cut-off between "lower" and "higher" intensity infections was taken as the median of all positive samples at baseline

References

1. Llewellyn S, Inpankaew T, Nery S, Gray D, Verweij J, Clements A, et al. Application of a multiplex quantitative PCR to assess prevalence and intensity of intestinal parasite infections in a controlled clinical trial. PLOS Negl Trop Dis. 2016;10(1): e0004380.
