# Supplementary material for: (S)WASH-D for Worms: A pilot study investigating the differential impact of school- versus community-based integrated control programs for soil-transmitted helminths
Source: PLoS Negl Trop Dis. 2018 May 3;12(5):e0006389. doi: 10.1371/journal.pntd.0006389 (PMC5933686; doi:10.1371/journal.pntd.0006389)
Supplement: S1 Table — (PDF) [file pntd.0006389.s004.pdf]

S1 Table. STH infections over time

Variable	Baseline		Follow-up			
	**Control** *n=372*	**Intervention** *n=110*	**Control** *n=303*	**Intervention** *n=107*	**DID**[a]	***P* value**
Ascaris spp. infections	48.7% (43.6–53.8)	7.6% (3.8–14.4)	23.4% (18.9–28.5)	0.9% (0.1–6.5)	18.6%	0.005
Ascaris spp. higher-intensity infections	27.3% (21.1–30.0)	0.9% (0.1–6.5)	4.3% (2.5–7.2)	0	22.1%	0.012
N. americanus infections	13.7% (10.6–17.6)	15.1% (9.4–23.3)	9.9% (7.0–13.8)	5.7% (2.5–12.1)	-5.6%	0.254
N. americanus higher-intensity infections	7.3% (5.0–10.4)	6.6% (3.2–13.3)	4.6% (2.7–7.5)	2.8% (0.9–8.5)	-1.1%	0.832
Ancylostoma spp. infections	1.1% (0.4–2.8)	0	0	0	1.1%	0.367
T. trichiura infections	2.2% (1.1–4.3)	1.9% (0.5–7.3)	2.0% (0.8–4.3)	0.9% (0.1–6.5%)	-0.8%	0.556

Results reported as: proportion (95% confidence interval).

[a] DID = difference in differences between intervention and control arms
