# Supplementary material for: (S)WASH-D for Worms: A pilot study investigating the differential impact of school- versus community-based integrated control programs for soil-transmitted helminths
Source: PLoS Negl Trop Dis. 2018 May 3;12(5):e0006389. doi: 10.1371/journal.pntd.0006389 (PMC5933686; doi:10.1371/journal.pntd.0006389)
Supplement: S2 Table — (PDF) [file pntd.0006389.s005.pdf]

S2 Table. Morbidity indicators over time

	Baseline		Follow-up			
	**Control**	**Intervention**	**Control**	**Intervention**	**DID[a]**	***P* value**
**Haematological parameters**	*n=381*	*n=116*	*n=324*	*n=112*		
Mean (SD) haemoglobin [g/L]	128.7 (11.2)	130.8 (10.2)	132.3 (10.9)	131.1 (10.9)	-3.3	0.085
Proportion anaemic (95% CI)	12.6% (9.6–16.3)	4.3% (1.8–10.0)	4.9% (3.0–7.9)	4.5% (1.9–10.3)	-7.8%	0.150
**Growth (all age groups)**	*n=382*	*n=124*	*n=356*	*n=116*		
Mean (SD) height-for-age *Z*-score	-2.00 (1.18)	-2.16 (1.10)	-1.89 (1.16)	-2.20 (1.09)	-0.15	0.067
Proportion stunting (95% CI)[b]	51.7% (46.6–56.8)	62.1% (53.2–70.2)	47.7% (42.3–53.2)	66.1% (56.9–74.2)	8.0%	0.096
Mean (SD) BMI-for-age *Z*-score[c]	-1.45 (0.83)	-1.93 (0.99)	-1.72 (0.96)	-2.01 (0.84)	0.19	0.554
Proportion thinness (95% CI)[d]	25.5% (21.3–30.1)	42.7% (34.3–51.6)	34.7% (29.7–40.1)	47.8% (38.9–57.0)	-4.1%	0.762
**Growth (age ≤10 years only)**	*n=225*	*n=86*	*n=206*	*n=83*		
Mean (SD) weight-for-age *Z*-score	-1.98 (1.00)	-2.42 (1.03)	-2.11 (0.92)	-2.61 (1.02)	-0.06	0.765
Proportion underweight (95% CI)[e]	53.3% (46.7–59.8)	65.1% (54.4–74.5)	58.4% (51.1–65.3)	76.8% (66.4–84.8)	6.6%	0.484

BMI = body mass index; CI = confidence interval; SD = standard deviation.

[a] DID = difference in differences between intervention and control arms.

[b] Stunting defined as greater than 2 standard deviations below the median height-for-age *Z*-score for reference population.

[c] BMI calculated as weight (kg) / height (cm)^2.

[d] Thinness defined as greater than 2 standard deviations below the median BMI-for-age *Z*-score for reference population.

[e] Underweight defined as greater than 2 standard deviations below the median weight-for-age *Z*-score for reference population.
